# Editorial

**Published:** 1869-06-01

**Authors:** 


### EDITORIAL.

The publication of the CHICAGO MEDICAL JOURNAL having passed into the hands of the subscriber, all payments for subscription and accounts, either past or future, must be made to me, unless otherwise directed. Subscribers who have neglected to pay their subscriptions on volumes previous to the present year must remit at an early day or the publisher will be compelled to discontinue sending them the JOURNAL.

Our subscribers will please remember the JOURNAL is $4.00 per annum, if not paid before July 1st of each year, and act accordingly.

The editorial management will remain as heretofore, in the hands of Dr. J. Adams Allen, assisted by Dr. W. Hay, to whom communications in his department should be addressed.

J. WOLCOTT HOOKER,
Room 12, Union Building.

CHICAGO, May 1st.

In order to give place to the reports of the proceedings of the Medical Congress at New Orleans, and of the Illinois State Medical Society, which present much that would interest our readers, we have been compelled to omit our usual original matter. We found ourselves troubled with an *embarras des richesses.*

